# Supplementary figures and images for: Pertactin contributes to shedding and transmission of Bordetella bronchiseptica
Source: PLoS Pathog. 2021 Aug 4;17(8):e1009735. doi: 10.1371/journal.ppat.1009735 (PMC8336816; doi:10.1371/journal.ppat.1009735)

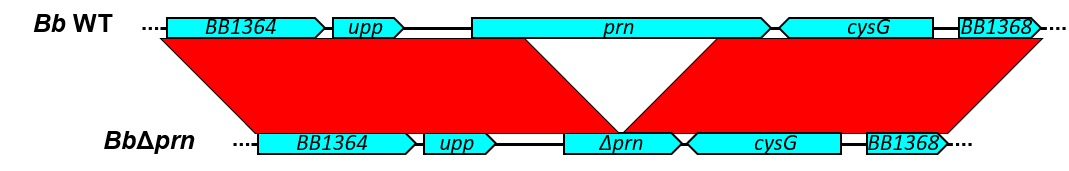

Supplement: S1 Fig — (TIF) [file ppat.1009735.s001.tif]

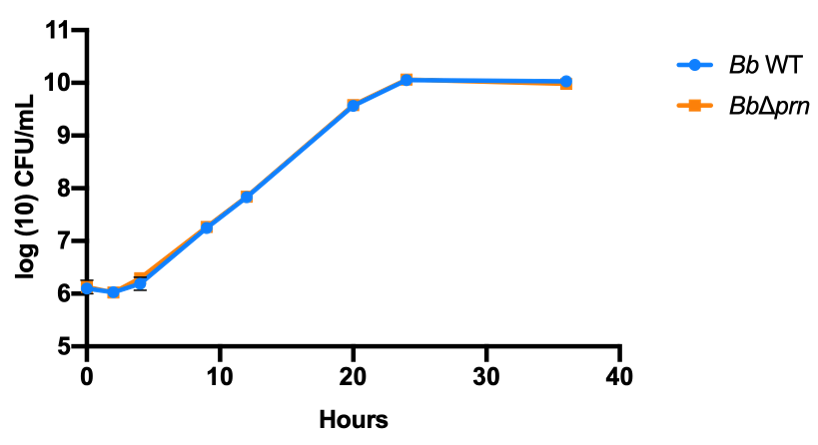

Supplement: S2 Fig — There were 3 replicates in each time point per group. Error bar shows the standard error of mean. Statistical significance was calculated using Two-way ANOVA. (TIF) [file ppat.1009735.s002.tif]

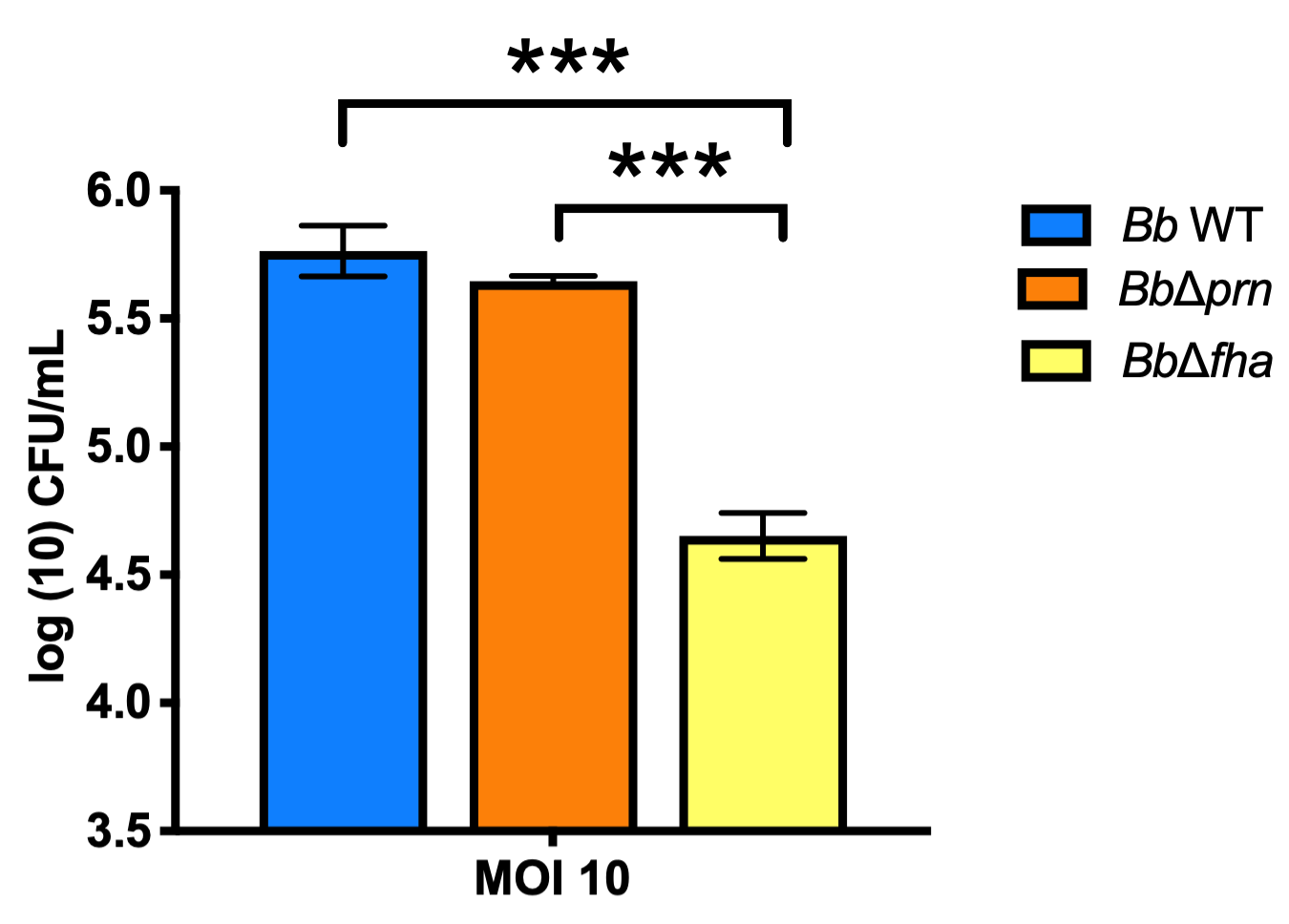

Supplement: S3 Fig — Adherence to A549 lung epithelial cells of Bb WT (blue), BbΔprn (orange) and BbΔfha (yellow) that was previously shown to be impaired in its ability to adhere to epithelial cells. There were 3 replicates in each group. Error bar shows the standard error of mean. Statistical significance was calculated using One-way ANOVA. ***p < 0.001. (TIF) [file ppat.1009735.s003.tif]

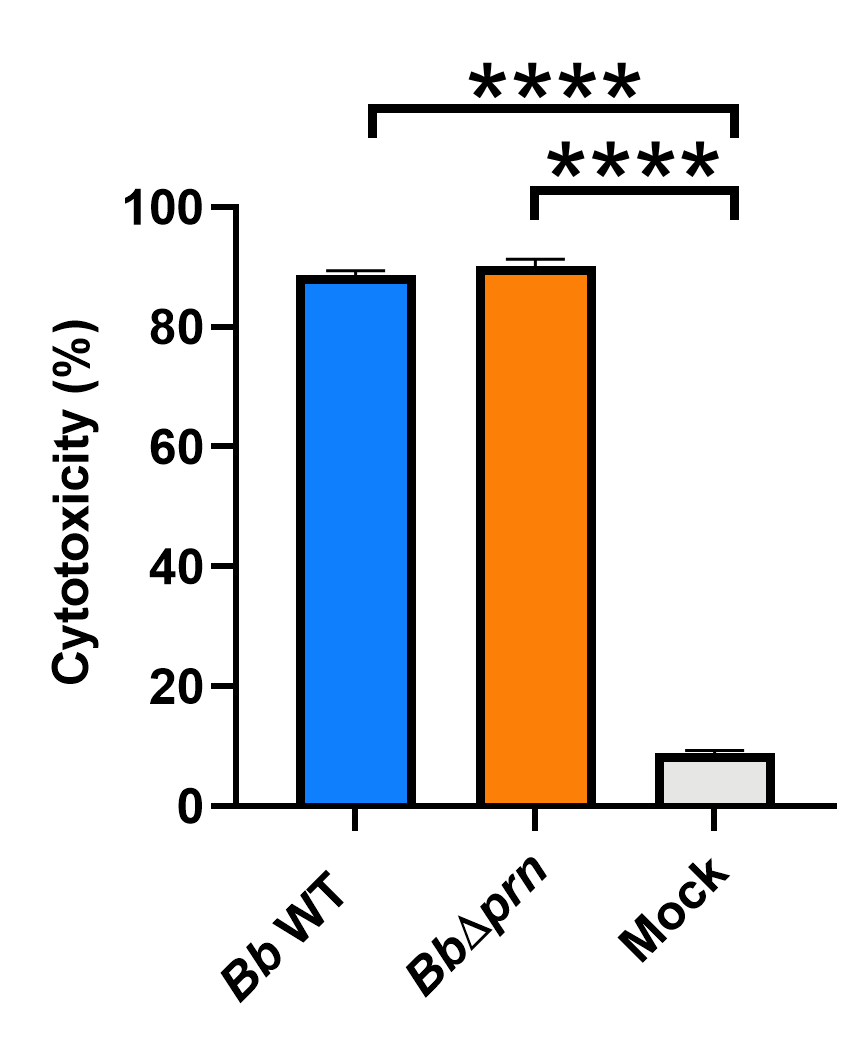

Supplement: S4 Fig — There were 3 replicates in each group. Error bar shows the standard error of mean. Statistical significance was calculated using One-way ANOVA. ****p < 0.0001. (TIF) [file ppat.1009735.s004.tif]

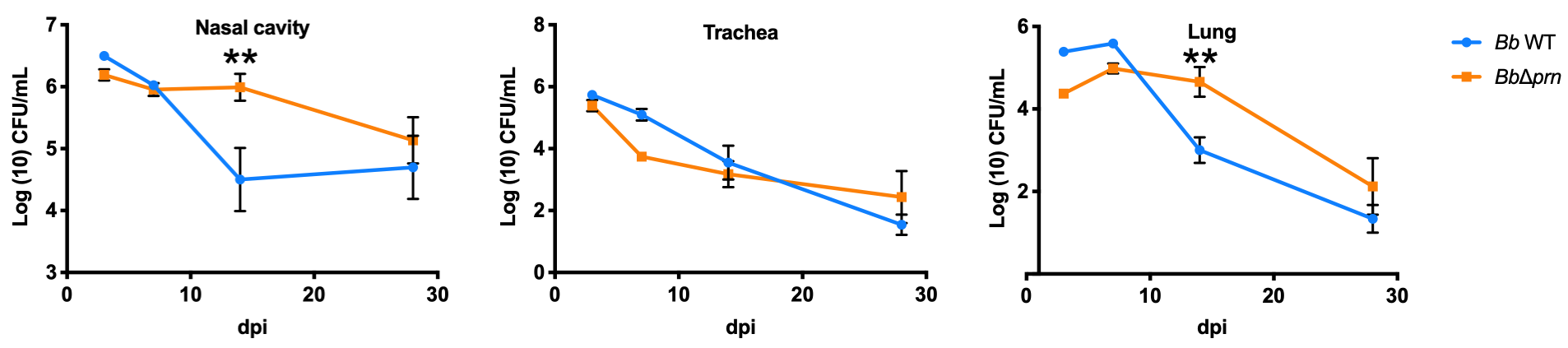

Supplement: S5 Fig — Bacterial CFU recovered on days 3-, 7-, 14-, and 28 dpi from the nasal cavities, trachea, and lungs of mice inoculated with either wild-type (blue) or mutant (orange) bacteria. There were 4 mice in each time point per group. Error bar shows the standard error of mean. Statistical significance was calculated by Two-way ANOVA. *p < 0.05, **p < 0.01, ***p < 0.001. (TIF) [file ppat.1009735.s005.tif]

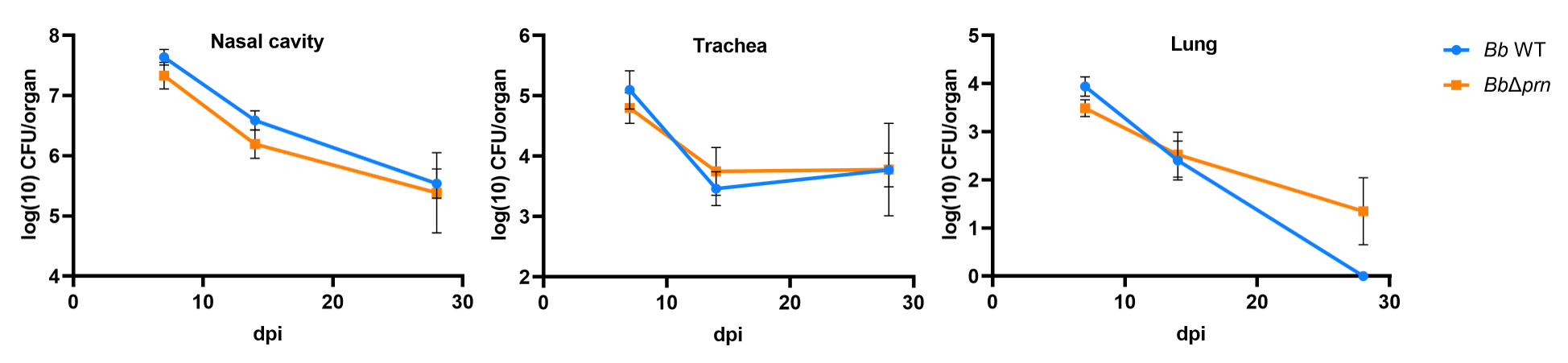

Supplement: S6 Fig — Comparative colonization profiles of Bb WT and BbΔprn in C3H/HeJ mice. Number of colony-forming units (CFU) recovered on days 3-, 7-, 14-, and 28 pi from the nasal cavities, trachea, and lungs of mice infected with either wild-type (blue) or mutant (orange) bacteria. There were 4 mice in each time point per group. Error bar shows the standard error of mean. Statistical significance was calculated by using Two-way ANOVA. (TIF) [file ppat.1009735.s006.tif]

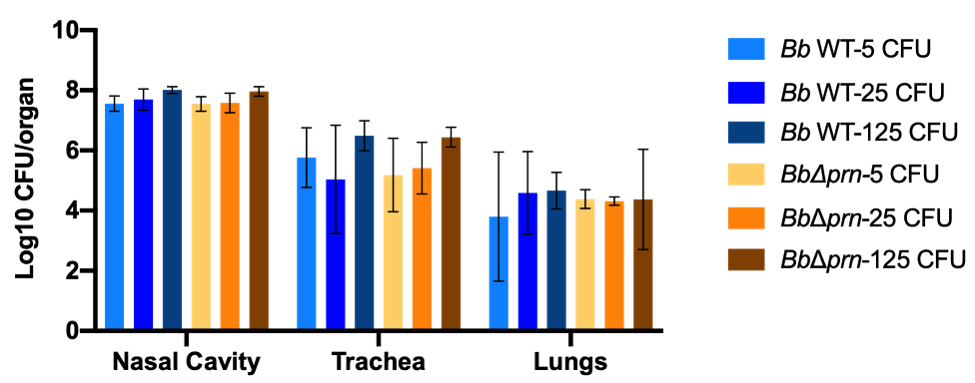

Supplement: S7 Fig — ID50 test of Bb WT and BbΔprn showing bacterial numbers at 7 dpi in respiratory organs of C3H/HeJ mice inoculated with incrementally increasing doses of 5 CFU, 25 CFU and 125 CFU. Number of CFU recovered from nasal cavities, trachea, and lungs revealed no difference in colonization between wild-type and mutant. There were 4 mice in each time point per group. Error bar shows the standard error of mean. Statistical significance was calculated by using Unpaired t-test. (TIF) [file ppat.1009735.s007.tif]

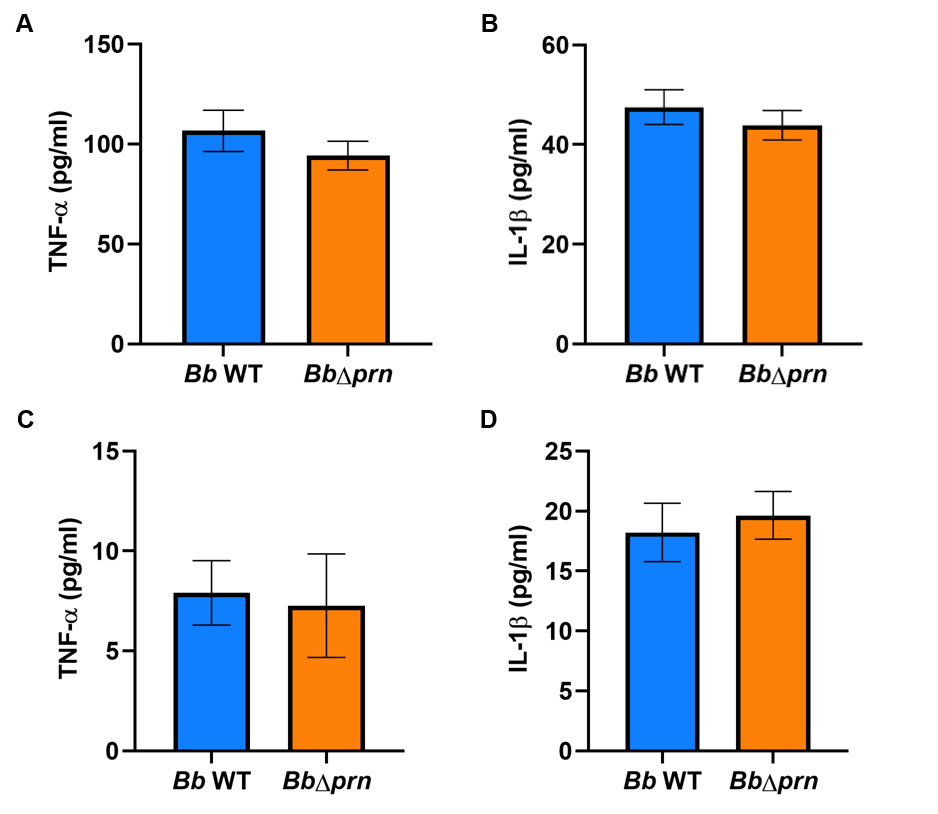

Supplement: S8 Fig — The levels of TNF-α in noses of mice infected with Bb WT (blue) or BbΔprn (orange) at 24hpi (A) or 48hpi (C). The levels of IL-1β in noses of mice infected with Bb WT (blue) or BbΔprn (orange) at 24hpi (B) or 48hpi (D). Error bar shows the standard error of mean. Statistical significance was calculated using unpaired T test. (TIF) [file ppat.1009735.s008.tif]

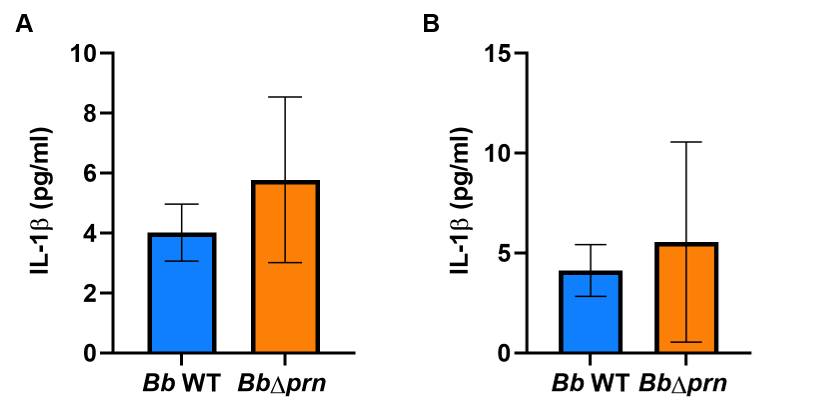

Supplement: S9 Fig — A) The IL-1β detected in the supernatant when RAW macrophages were exposed with Bb WT (blue) or BbΔprn (orange) with a MOI at 10 for 1 hour. B) The IL-1β detected in the supernatant when RAW macrophages were exposed with Bb WT (blue) or BbΔprn (orange) with a MOI at 100 for 1 hour. Error bar shows the standard error of mean. Statistical significance was calculated using unpaired T test. (TIF) [file ppat.1009735.s009.tif]

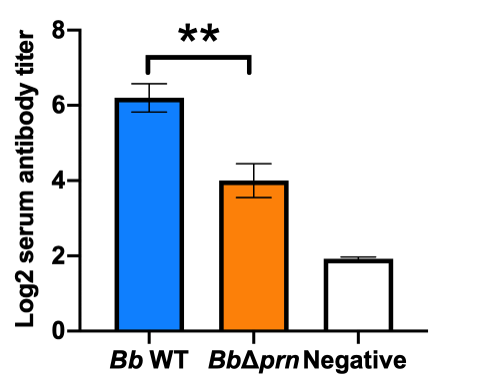

Supplement: S10 Fig — IgG antibody titers against B. bronchiseptica were determined in sera of C3H/HeJ mice infected with either Bb WT (blue) or BbΔprn (orange) at 28 dpi. There were 4 mice per group. Error bars show the standard error of mean. Statistical significance was calculated by using One-way ANOVA. *p < 0.05, **p < 0.01, ***p < 0.001. (TIF) [file ppat.1009735.s010.tif]

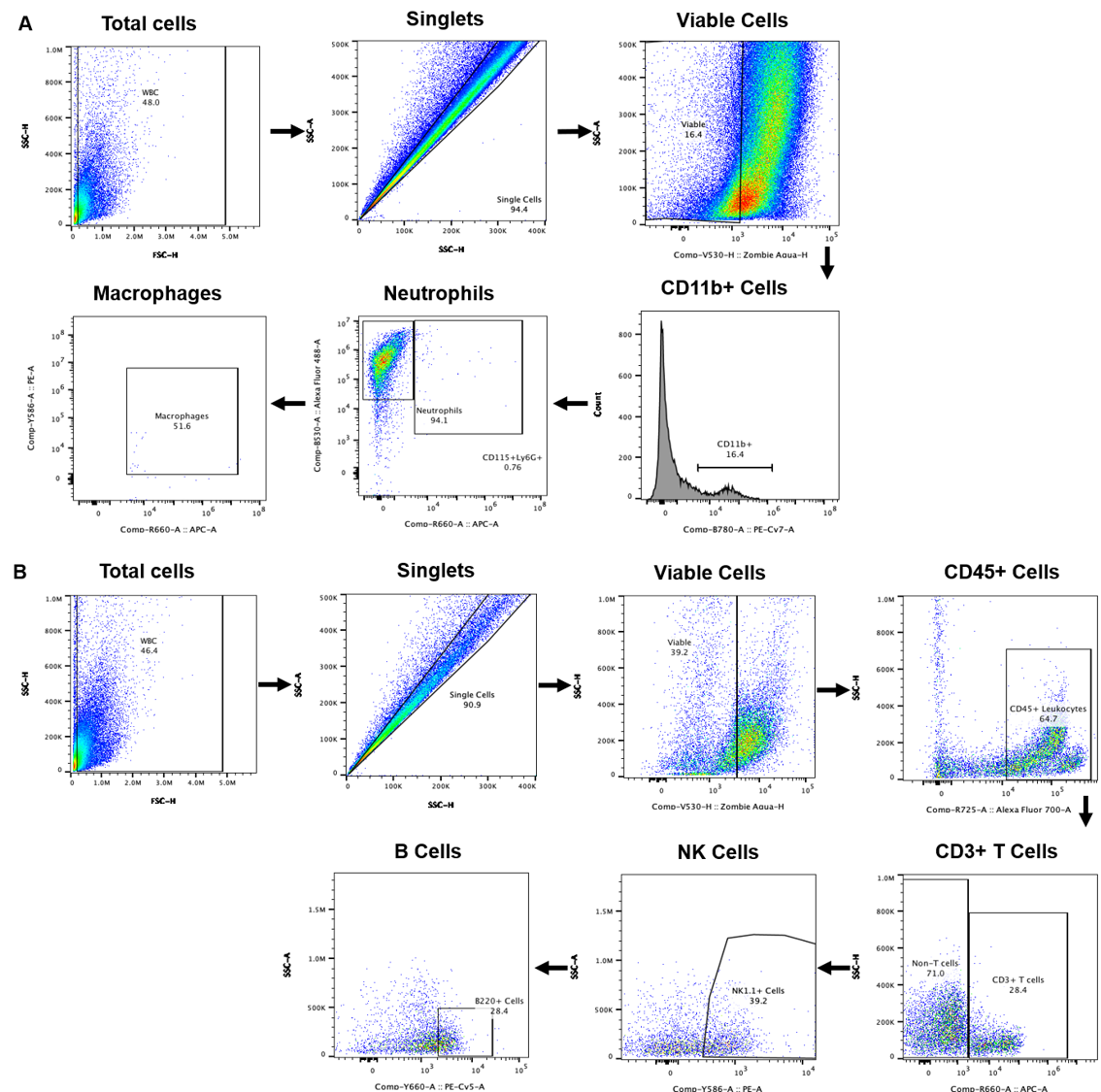

Supplement: S11 Fig — A) Gating strategy for myeloid cell types. B) Gating strategy for lymphoid cell types. (TIF) [file ppat.1009735.s011.tif]

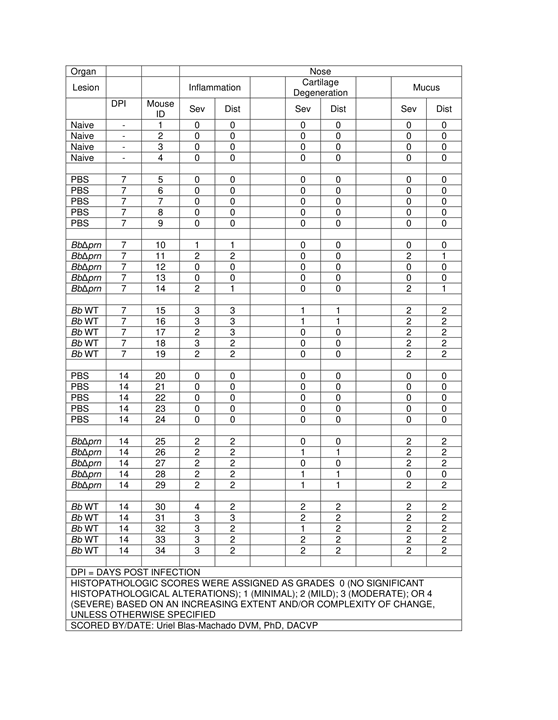

Supplement: S1 Table — (TIF) [file ppat.1009735.s012.tif]
