# Supplementary material for: Pertactin contributes to shedding and transmission of Bordetella bronchiseptica
Source: PLoS Pathog. 2021 Aug 4;17(8):e1009735. doi: 10.1371/journal.ppat.1009735 (PMC8336816; doi:10.1371/journal.ppat.1009735)
Supplement: Attachment — Submitted filename: Response to reviewers.docx [file ppat.1009735.s013.docx]

**Editors**

While this manuscript does provide some novel findings the manuscript as it currently stands does not reach the threshold for publication in PLOS Pathogens. Increased mechanistic insight is required to better prove the role of Prn and most importantly a better attempt must be made to translate the relevance of the model to B. pertussis infection in humans.

To understand mechanisms underlying the role of pertactin in bacterial shedding and transmission, we have now extended our study on the role of pertactin by testing the effects of purified PRN on inflammasome activation, analyzing immune cell types recruited by PRN using flow cytometry, and verifying the role of pertactin in bacterial dissemination and intracellular survival within the mouse model.

**Reviewer # 1**

**Summary**: There are some novel and potentially interesting findings in this study, but unfortunately it is very thin, lacking detail and mechanistic insight. The authors need to do a good deal more work before this story is complete.

To address the comments from the reviewers, we have now extended our study on the role of pertactin by testing the effects of purified PRN on inflammasome activation, analyzing immune cell types recruited by PRN, and verifying the role of pertactin in bacterial dissemination and intracellular survival within the mouse model.

**Major issues**

**1.** The authors justify their research on pertactin (PRN) from Bordetella on the basis of the observations that pertactin-deleted mutants of Bordetella pertussis have emerged in populations immunized with acellular pertussis vaccine that include PRN as an antigen. However, they carry out their research with B. bronchiseptica and the justification is that they have a mouse transmission model with this bacterium, which they don’t have with B. pertussis. However, many aspects of the study, local inflammation, mucus secretion and intracellular survival could have been done with B. pertussis, where they could have used human clinical isolates, rather than lab-generated mutants of B. bronchiseptica.

We are familiar with the allure of working with the actual human pathogen. However, our work, and that of many other outstanding researchers, has clearly demonstrated the value of working with model systems that use *natural* host-pathogen interactions. The *B. bronchiseptica*-mouse system continues to reveal important aspects of interactions relevant to pertussis as a disease and that happen in the course of a natural infection. Other groups have performed studies like those this reviewer requests, using the human pathogen in mice it does not normally infect, but struggled to find a relevant biological function as important as that we have revealed here. We view such experiments as having their own caveats in interpretation. Amongst the strengths of our findings is the confidence that the observed functions are observable in the context of natural host-pathogen interactions. We respectfully leave others to consider the multiple iterations of less natural combinations of bacteria and host. We consider those to be problematic and therefore of lower priority.

**2.** The biggest issue with this report is that it lacks depth, the essential data could be condensed into 2 figures. Do we need a whole figure showing only two bars on a bar graph (Fig. 3) These bars don’t have error bars, making less of case for including them in a figure at all. The first two figures of negative data (Fig 1 and 2) that could be one supplementary figure. The 3 interesting observations are a) transmission and shedding is lower in mice infected with the PRN-neg mutant, b) there is less inflammation/mucus secretion in nasal cavity of mice infected with the PRN-neg mutant c) intracellular survival in macrophages in vitro is lower with the PRN-neg mutant. However, none of these observations are linked. The authors speculate on immune responses being stronger in mice infected with WT compared with PRN-negative mutants of B. bronchiseptica, but have not examined this. They speculate about higher inflammasome activation with WT bacteria, but have not assessed this. There is not data linking either intracellular survival or inflammation with transmission.

We believe that most readers will see the links between increased Pertactin-mediated inflammation and mucus production in the nose and understand how those contribute to shedding and therefore to transmission.

a. We have reorganized the results as suggested.

b. We have added an analysis of cell types in nasal cavity of mice infected with either WT or mutant strains by flow cytometry and results are shown in Figure 4.

c. We have tested inflammasome activation in macrophages induced by either WT or mutant strains. The results, shown in Figure S10**,** demonstrate that macrophages exposed to pertactin deletion mutant strain have higher IL-18 secretion compared to the groups exposed to the WT strain, which coincides with the previous studies the reviewer mentioned in question NO. 3. The apparently contradictory observations suggest that the inflammation induced by macrophage-like cell line, in an artificial and extraordinarily dense in vitro infection model, may not be relevant during infection.

3. The authors should consider some studies with purified PRN where they look at its effect on inflammatory responses of macrophages in vitro. There is some evidence that PRN works with FHA to suppress LPS-induced inflammatory responses by macrophages. It is not clear how those observation would fit with the suggestion in the current study that PRN may promote inflammation. Either way, it would worth testing the effect of PRN on induction or inhibition of inflammasome activation? Studies such as these might help to expand on their theories around the inflammasome.
We have now carried out experiments to test inflammasome activation in macrophages which were exposed to either purified PRN or BSA added to the cell culture medium at concentrations of 40 nM (ref PMID: 29746836) or 120nM. After 24-, 48- and 72-hours culture, cell culture supernatants are collected for IL-1β and IL-18. The ELISA results show that there is no detectable IL-1β and IL-18 in these samples. The possible explanations are that there were undetectable low level inflammasome activation or the inflammation observed in mice involve some other immune cell types besides macrophages, or the caveats of using macrophage-like cell lines *in vitro.*

4. The B. pertussis challenge dose for the early figures is 0.5 million CFU, but is only 150 CFU in the later studies, how can these be reconciled or compared in making conclusion on the course of infection versus inflammation or shedding etc.

0.5 million CFU delivered deep into the lungs is the conventional pneumonic mouse model. We have clarified in several places now that we are using much lower doses (~150 CFU) to allow more direct and quantitative measures of aspects of colonization, early growth, shedding and inflammation in the context of a more natural course of infection.

**Minor issues**

The preparation of manuscript, especially the figures, are not up to the standard required for a top-class journal. e.g. out of sequence figure call out, references to figures in the Introduction and discussion, poor quality figures; it is almost impossible to read the font on some figures. The Y axis scales on panels within figures are not consistent. Some Y axes are split when they don’t need to be, and many don’t start at zero.

I think many people in the field would disagree with the statement in the introduction that whole cell vaccine had “relatively minor side effects”.

We have improved the quality and consistency of figures in the manuscript. We have replaced “relatively minor side effects” with “side effects”.

**Reviewer #2**

**Summary**

The paper addresses a long standing query in the Bordetella field: the true role of Prn in infection biology. Early studies suggested Prn to be an adhesin, and the presence of an RGD motif supports this. However, clear evidence for this has been obtained from in vitro studies only, and in vivo studies demonstrated efficient colonisation in the absence of Prn.

Here, careful studies using very small inoculae very clearly demonstrate that BB prn mutants can very efficiently colonise and persist in the respiratory tracts of mice, demonstrating that Prn is not required for these processes in BB.

Instead, data is presented to argue for a role in intracellular survival, and in inducing inflammatory responses to BB infection. Of particular interest is the demonstration of reduced shedding of BB prn mutants from the respiratory tract and reduced transmission between index and recipient mice. The authors combine these findings to suggest a role for Prn in inducing inflammation, that facilitates bacterial shedding and onward transmission.

This story makes a telling contribution to defining the infection biology of the bordetellae, and adds to understanding the role of Prn in this. Prn is of particular importance as it is one of the components of many of the acellular Pertussis vaccines. Importantly, in countries experiencing resurgence of pertussis, there is a stark increase in the frequency of isolates that are Prn-deficient, and there are multiple lines of evidence to suggest that Prn-deficient B. pertussis strains have a fitness advantage in populations that are highly vaccinated with the acellular vaccines.

In the light of this, the study is important, as it addresses the role of pertactin in infection which is crucial to understanding the consequences of Prn deficiency for B. pertussis fitness.

**Major issues**

A key message is that Prn contributes to the induction of inflammation by BB. As it stands, it is not possible to determine if the data supports this.

We have enumerated immune cell types and numbers in nasal cavities of mice which were inoculated with either *Bb* WT or pertactin deletion mutant strain by flow cytometry. The results show that much higher numbers of neutrophils, NK cells and B cells are recruited in nasal cavity by the WT bacteria expressing pertactin (Fig 4). In addition, histopathology results show that more mucus is secreted in nasal cavities of mice inoculated with the WT strain compared to mice inoculated with the *prn* deletion mutant strain.

Ln342-351. More detail is required for the approach to histopathology scoring. Currently, it is not possible to understand how scores were determined. For example, grade 0- no significant alterations. What counts as significant? Grade 1 – minimal, which would be zero? Scoring based partly on ‘complexity’ of change. What does this mean? What area of the total tissue was scored? How was bias excluded from the selection of areas. Were observers blinded to the source in terms of WT or mutant? I appreciate that drawing quantitative measures from these sorts of data is difficult, but there needs to be greater clarity as to how quantitative measures were drawn from visual images for the conclusion regarding the role of Prn in inflammation to stand up to scrutiny.

We have now included the scoring standards as supplementary data (Figure S8), clarifying the basis for scores.

**Minor issues**

There is a clear contradiction between Prn contributing to transmission and the striking increase in Prn-deficiency among B. pertussis. The authors mention possible compensation for loss of Prn from B. pertussis by other autotransporters, but there is no clear rationale for this. Could the authors comment on the difference between BB and B. pertussis in the ris system that has been linked to intracellular survival, and for which Chen and Stibbitz suggested a role in promoting the expression of Vrgs and aerosol transmission (Curr Opin Microbiol. 2019. DOI: 10.1016/j.mib.2019.01.002. The authors should also acknowledge that their findings could indicate that Prn has a different role in B. pertussis compared to BB. This does not question the findings reported here regarding the role for Prn in BB infection, but if a key motivation for this study was to address the question of Prn and Prn-deficiency among B. pertussis, then a more rounded discussion of this is required.

We have added a discussion on the difference between *B. pertussis* and *B. bronchiseptica* and added the following papers in the reference section: PMID: 11470358; PMID: 9746560; PMID: 30870653.

We have also added a discussion on the possible different roles of PRN in *B.bronchiseptica* and *B.pertussis.*

There are a number of other points that the authors could consider:

Ln 117. Describing BB and BP as 98% identical at the nucleotide level is misleading. Speciation of B. pertussis involved loss of over 1Mb of DNA compared to BB, describing identity as 98% infers there is only 2% difference between the two.
We will correct it as “shared genes are ~ 98% identical at the nucleotide level”.

Ln119. ‘ref5’ suggests a citation issue.

We have corrected this. Thank you!

Ln131. It is unusual to refer to results figures in the introduction.

We have removed the reference to a figure from the introduction.

Ln209-212. This is discussion not results.

We have deleted discussion text from the results.

Ln204. States RAW cells were infected with bacteria at an MOI of 10 and 1, materials states 100, 10 and 1. States extracellular bacteria were killed by 1 and 2 hours of gent treatment. But in Ln276-8, it suggests Prn mutant is gent resistant? Ln 315 states extracellular bacteria were killed with polymyxin but this was added 1 hour after addition of bacteria to the RAWs. There appears contradiction between these sections.
We have corrected these mistakes! Thank you!

All of the comments are aligned with one hour exposure to permit macrophage phagocytosis of bacteria, after which the culture was treated with antibiotics for different time periods.

If polymyxin B was used, is the sensitivity of WT and the Prn mutant to PMB identical, particularly at low levels? Some PMB will cross the RAW membranes and an increased sensitivity of the Prn mutant would reduce numbers recovered. The cytotoxicity of BB for mammalian cells could compromise cell membranes. The same applies for Triton X-100 as this was used to lyse the RAW cells to recover the intracellular bacteria. It is important to show that different sensitivity was not the reason for the difference in recovered bacteria.
Thank you for this excellent suggestion. Since the mutant strain is resistant to gentamicin, we are unable to compare intracellular survival between *Bb* WT and *Bb*Δ*prn* by using the gentamicin protection assay. After multiple attempts with polymyxin, we are unsatisfied with the behavior of this assay. In view of this we have removed the intracellular survival data from the manuscript. Recovery of *Bb* WT, but not the prn mutant, from spleens of infected mice suggests that *Bb* WT may have a Pertactin-dependent capacity to get to or survive within the spleen. This is a complex phenotype that will require extensive examination in subsequent studies.

Figure 1. For transmission, index mice were co-housed with naïve mice for 21 days. Wt mice were cleared from the lungs of the mice sometime between the day 14 and day 28 time points. It is not clear when these WT were cleared, it might have been day 27, or day 15. Prn mutants were recovered from the lungs on day 28. Thus the colonization profiles of WT and prn mutants are different, although not in the region where shedding would be expected to occur from (nasal cavity), and this wouldn’t explain reduced shedding of Prn mutants, but it’s not correct to say no difference.

We have improved figures and error bars. Since there is no statistically significant difference seen in bacterial numbers recovered from lungs at day 28, we retain the statement that there is no difference in the colonization of respiratory tract.

Figure 2. It is very difficult to be precise in getting exactly 5 cfu per inoculum. Were the inoculae plated? If so, show data to show the likely range of inoculate the mice will have received for each of the 3 categories. The data suggest that none of the mice received zero cfu?
To test ID50, we had more than three groups of mice with a wide range of inocula. After plating out the inocula, we know the average number delivered for each group and that those number are quite consistent. If they were not, we would occasionally observe an inoculated animal that is not colonized, and we have not.

Ln189, In the text, Figure 5A and B are identified as showing histopathology induced by WT BB, but in the Fig, A-C are panels showing sections from control mice. This might stem from the odd numbering for Figure 5 which appears to be 3 quite separate figures, but are included in one Figure labelled at 5A-C. However, 5A then has panels A-I, so creates an odd labelling of 5AA, 5AB but this is not used in the text.
We have corrected the mistakes in the figure 2. Thank you!

Fig 5A. It is notoriously difficult to demonstrate a clear difference between pathology using single panels from single samples. As mentioned above, some explanation of how histopathology was scored, and how cell sections were selected for analysis in an unbiased way. It is difficult for the reader to understand the data depicted in Figs 5B and C without this.
We have now added scoring standard in supplementary data. (Supplementary Figure S8.)

Figure 6. For the intracellular survival of BB in RAWs, the data is presented as a survival ratio, but there is no explanation of what this is, or how it was calculated. In Fig 6A data is shown for WT and Prn mutants at MOIs of 1 and 10 for 2 hours post internalization. In Fig 6B data is shown for 1, 2, 4 and 24 hours post internalisation, but the data for 2 hours appear to be different to that in Fig 6A. Are these data from a different experiment, or a different MOI?

We have now clarified that the survival ratio is the number of CFUs recovered / bacterial CFUs of the inoculum.

The results are from the same experiment. Figure 6A is with two MOI, 10 and 1, for 2 hours post internalization. Figure 6B is with MOI as 10 for different hours post internalization. We have changed the Y axis in Figure 6A to make it easier to compare various groups.

**Reviewer #3**

**Summary**

Using a TLR4 deficient mouse model, Ma et al explore the role of Pertactin (PRN) of B. bronchiseptica in colonization, growth, spread within the respiratory tract, shedding, and transmission. It was demonstrated that while PRN deletion mutants showed no change in colonization of the trachea and lungs of mice compared to the WT strain, shedding and transmission was significantly reduced. The respiratory epithelia of mice infected with the PRN deletion mutant exhibited significantly reduced inflammation and mucus accumulation compared to the WT strain. Although the results suggest an important role for pertactin in B. bronchiseptica, the authors overstate what their model shows with regards to B. pertussis infections.

The argument that studying the B. bronchiseptica in one of its natural hosts provides valuable insights into B. pertussis infections in humans would only be valid if the diseases caused by the two pathogens in their natural hosts were comparable. Although the bacteria are genetically similar and share some virulence factors, the diseases caused by B. bronchiseptica and B. pertussis are very different, with different tropisms (humans/NHP vs. many mammals except humans) and clinical presentations (acute/weeks vs prolonged/chronic). Additionally, pertussis toxin is a key toxin and essential virulence factor in pertussis infections (PT-only vaccines prevent disease), yet it is not expressed by B. bronchiseptica—highlighting a major difference in disease mechanisms. Bb and Bp also express different lipid A structures and polysaccharide chains (LPS vs LOS), with Bb having a more inflammatory lipid A/LPS (2). Using a TLR4-deficient mouse strain to allow disease transmission further confounds the applicability of these results to B. pertussis infections, since TLR4 appears to have different roles in Bb and Bp infections (3). PRN’s contribution to inflammation, which the researchers suggest may influence mucus production, shedding and transmission, may be insignificant in a TLR4-competent mouse (a key innate immune recognition molecule for endotoxin and host DAMPs). It is possible the effects observed are artifacts of the experimental model.
B. pertussis survives very poorly outside the host whereas B. bronchiseptica has been shown to survive for prolonged periods of time in water. B bronchiseptica can be efficiently transferred by contact and by sharing of water sources. This is not true of B pertussis which is transmitted by respiratory droplets and aerosols.

We modestly and respectfully quote George E. P. Box - “All models are wrong, but some are useful”. We, and others, are highly excited about the opportunity to understand the mechanistic basis for transmission and to understand what Pertactin CAN do in this experimental system. Its relevance to B. pertussis-human transmission may be debated, as we in fact objectively discuss in the manuscript. But the argument that this experimental system is not relevant, useful and informative because the two pathogens induce different diseases is ignoring a mountain of similarities to focus on relatively minor differences. Applied to other pathogens, this statement would discount some of the most informative and useful research on close relatives of human pathogens that don’t efficiently infect mice. We do not consider it necessary or productive to perform a deep dive to refute these claims that are out of touch with the scientific mainstream, and the other reviewers. Instead, we will rely on the objectivity of the editor, and a brief look at the literature and the many articles using this and other such experimental systems published in the best peer reviewed journals.

Other comments in this review were quite helpful, and for that we are grateful.

**Major Issues**

1. The authors state that RB50 transmits efficiently between mice (lines 143-144) but that only appears to be the case in an immuno-deficient mouse strain. In order to draw conclusions about the transmission of PRN-negative B. bronchiseptica strains, the authors should explore animal models of B. bronchiseptica transmission that don’t require the use of immunodeficient animals (e.g. the pig model)

Thank you for this suggestion, however, the prohibitive costs of establishing and conducting experiments using large animal models is a major limitation.

2. The researchers should complement or repair the isogenic PRN KO, to confirm that unintentional, off-target effects are not contributing to their disease models (mice, cell culture).

The same mutant strain has been described and been used in a published paper, PMID:20421378. In this paper, the phenotype was fully restored to that of wild type when prn mutation was complemented. In our study, to allow mutant and WT to be distinguished in “competition assays”, gentamycin resistance cassette was added following the promoter of FHA. Additionally, for further confirmation, we performed whole genome sequencing of the mutant strain. The results showed only the pertactin gene was deleted.

3. The researchers should consider assessing inflammasome activation in their in vitro intracellular invasion system as they speculate that may explain differences in inflammation induced by the WT and PRN-deficient strains.

We tested inflammasome activation in macrophages induced by either *B. bronchiseptica* WT or the mutant strain. The results are summarized in Figure S10**.** The results show that macrophages exposed with the pertactin deletion mutant strain secreted higher levels of IL-18 compared to the WT strain. These observations agree with the previous studies (referred to by reviewer 1). However, the discrepancy between results of our in-vitro assay and our histological observations using the mouse model, indicate that the pro-inflammatory response observed with macrophages in vitro may be playing a less significant role in the whole-body immune response.

**Minor issue**

1. Researchers did not indicate if their p-values for student T tests were corrected for multiple comparisons, which may impact the statistical validity of the results. It is possible that more mice are needed to reach statistical significance, and indeed may allow for some statistical comparisons between WT and PRN-KO mice in terms of dissemination to the spleen at day 7 and 14. Animal numbers and error bars (SD? SEM?) should be described in the figure legends.

We have clarified statistics in all the figures.

We have increased mouse number for the bacterial dissemination test. The results are shown in Figure 5.

2. The statement that natural B. pertussis infections begin in the nasopharyngeal region, progress slowly during a prolonged catarrhal stage and do not normally even reach the lungs (lines 63-65). Should be deleted or supported by primary references. The natural progression of B. pertussis colonization in humans is unknown and has not been studied. The data that’s available from human autopsy cases and NHP studies suggests B. pertussis penetrates deeply into the airway.
We have now cited the CDC page and based our description on theirs. Human autopsy samples are clearly extreme outliers of the last stage of terminal disease, and cannot be used to understand initial and early steps in the preceding catarrhal stage which the literature and CDC says is a nasopharyngeal infection. *B. pertussis* rarely kills, but NHP studies purposely induced severe lung disease with near lethal doses by inoculating huge numbers of bacteria deep into the lower respiratory tract. We present our low dose nasopharyngeal inoculation approach as a more natural alternative to those extreme inoculation conditions, and demonstrate that our approach leads to a more natural course of infection. The data speak for themselves in this regard.

3. Line 235: “Bb WT inoculated mice shed significant more bacteria than BbΔprn inoculated mice” cite Fig. 4
We have corrected this. Thank you!

2. Line 247-249: “In addition, Bb WT showed significantly higher intracellular survival in macrophages compared with the PRN deletion mutant in an in vitro assay” cite Fig. 6

We have corrected this. Thank you!

3. Line 255-257: “Thus, increased intracellular survival may result in a stronger immune response against Bb WT than BbΔprn, consistent with the histopathology analysis in this study” cite Fig. 5

We have corrected this. Thank you!
