# Supplementary material for: Pertactin contributes to shedding and transmission of Bordetella bronchiseptica
Source: PLoS Pathog. 2021 Aug 4;17(8):e1009735. doi: 10.1371/journal.ppat.1009735 (PMC8336816; doi:10.1371/journal.ppat.1009735)
Supplement: Attachment — Submitted filename: Response to reviewers.docx [file ppat.1009735.s014.docx]

**Summary of changes**

1. To address the issues raised by reviewers on using *B. bronchiseptica* for modeling *B. pertussis*, we have now modified the manuscript (Introduction section) to highlight the major differences between *B. pertussis* and *B. bronchiseptica* (Lines 102-108).

2. To explore the possible mechanisms behind the function of pertactin on bacterial shedding and transmission, we measured the levels of the pro-inflammatory cytokines, TNF-α and IL-1β, produced in the nasal cavities of mice inoculated with *Bb* WT or the pertactin mutant at days 1 and 2 post inoculation. We have also tested the secretion of these two cytokines by RAW macrophages when exposed to *Bb* WT or mutant strain at different MOIs. The inductive functions of pertactin on TNF-α, a cytokine for neutrophil recruitment and priming, may explain the higher inflammation we observed in nasal cavities of *Bb* WT infected mice (Lines 223-231).

3. To test whether PRN induces inflammasome activation, we tested IL-1β and IL-18 secretion by RAW macrophages when exposed to *Bb* WT or mutant strain. Assays were done using fresh kits from R&D Systems. The IL-18 levels were similar when macrophages were exposed with either *Bb* WT or mutant, so we removed speculation about inflammasome activation from the manuscript (Lines 248-255 and S9 Figure).

**Questions:**

1. In particular, the lack of mechanistic data to explain the role of PRN in inflammation. The reviewers had suggested assessing immune responses and inflammasome activation induced by WT versus PRN- mutant bacteria to explain the higher inflammation with the WT bacteria and although you have looked at immune cell recruitment in the respiratory tissue, which is useful, this is very superficial and not a readout of immune response. The inflammasome activation theory has not been supported by any data. Indeed, the new data suggests that the mutant bacteria induce more, not less, IL-18 production from macrophages which you suggest may be due to the cell type chosen. If this is the case, then perhaps an alternative cell type should be considered.

To test possible inflammasome activation based on reviewer’s comments, we used fresh ELISA-based assay kits to evaluate secretion of IL-1β and IL-18 in relation to PRN. When RAW macrophages were exposed to *Bb* WT or mutant strain at an MOI of 10 for 4 hours, similar levels of IL-1β and IL-18 were detected in the supernatants of the cell cultures (Figure 1 below). The results suggested that PRN is not involved in inflammasome activation.

**Figure** **1. PRN does not induce inflammasome activation**. A) Similar levels of IL-1β were detected in the supernatants when Raw macrophages were exposed with *Bb* WT or mutant for 4 hours. B) Similar levels of IL-18 were detected in the supernatants when Raw macrophages were exposed to *Bb* WT or mutant for 4 hours.

2. Secondly the lack of data with purified PRN. In your response you mention an experiment to look at inflammasome activation via IL-1 and IL-18 production in the reply to reviewers but show no data because the experiment didn’t seem to work. This is may be because two signals for inflammasome activation have not been used. It will be important to definitively prove or disprove the theory on inflammasome activation. Overall the insights into the biological action of pertactin are interesting but it's difficult to draw conclusions about the role of pertactin in B. pertussis infections since the rapid loss of pertactin from circulating strains indicates it is not required for infection or transmission of B. pertussis within the human population.

We used fresh ELISA kits to detect the secretion of IL-1β and a more sensitive ELISA kit to detect IL-18 when macrophages were exposed to purified PRN (40mM) (ref PMID: 29746836). The results showed that purified PRN did not affect IL-1β secretion and thus did not induce the inflammasome, but increased secretion of the inflammatory cytokine IL-18 (Figure 2 below), even though the effect was not statistically significant due to relatively large standard errors.

**Figure 2. Purified PRN does not induce inflammasome activation.** A) Similar levels of IL-1β were detected in the supernatants when Raw macrophages were exposed to 40mM purified PRN. B) Elevated levels of IL-18 were detected in supernatant when Raw macrophages were exposed with or without 40mM purified PRN without statistical significance.

3. Finally, while it is agreed that models do not have to recreate every aspect of a disease system to be useful, but in order for a model to be useful, it’s strengths and weaknesses have to be considered and discussed. In the introduction for example the significant differences between B. bronchiseptica and B. pertussis infections are somewhat glossed over while emphasizing the similarities between the two strains.

We have listed out the major difference between *B. bronchiseptica* and *B. pertussis* in the introduction and in the discussion, stated the weaknesses of using *B. bronchiseptica* as a model for *B. pertussis* study, and expressed the need for new models (Lines 102-108 and 319-321).
